# DIGItal Health Literacy after COVID-19 Outbreak among Frail and Non-Frail Cardiology Patients: The DIGI-COVID Study

**DOI:** 10.3390/jpm13010099

**Published:** 2022-12-31

**Authors:** Marco Vitolo, Valentina Ziveri, Giacomo Gozzi, Chiara Busi, Jacopo Francesco Imberti, Niccolò Bonini, Federico Muto, Davide Antonio Mei, Matteo Menozzi, Marta Mantovani, Benedetta Cherubini, Vincenzo Livio Malavasi, Giuseppe Boriani

**Affiliations:** 1 Cardiology Division, Department of Biomedical, Metabolic and Neural Sciences, University of Modena and Reggio Emilia, Policlinico di Modena, 41125 Modena, Italy; 2 Clinical and Experimental Medicine PhD Program, University of Modena and Reggio Emilia, Policlinico di Modena, 41125 Modena, Italy

**Keywords:** atrial fibrillation, frailty, COVID-19, digital devices, telemedicine

## Abstract

Background: Telemedicine requires either the use of digital tools or a minimum technological knowledge of the patients. Digital health literacy may influence the use of telemedicine in most patients, particularly those with frailty. We aimed to explore the association between frailty, the use of digital tools, and patients’ digital health literacy. Methods: We prospectively enrolled patients referred to arrhythmia outpatient clinics of our cardiology department from March to September 2022. Patients were divided according to frailty status as defined by the Edmonton Frail Scale (EFS) into robust, pre-frail, and frail. The degree of digital health literacy was assessed through the Digital Health Literacy Instrument (DHLI), which explores seven digital skill categories measured by 21 self-report questions. Results: A total of 300 patients were enrolled (36.3% females, median age 75 (66–84)) and stratified according to frailty status as robust (EFS ≤ 5; 70.7%), pre-frail (EFS 6–7; 15.7%), and frail (EFS ≥ 8; 13.7%). Frail and pre-frail patients used digital tools less frequently and accessed the Internet less frequently compared to robust patients. In the logistic regression analysis, frail patients were significantly associated with the non-use of the Internet (adjusted odds ratio 2.58, 95% CI 1.92–5.61) compared to robust and pre-frail patients. Digital health literacy decreased as the level of frailty increased in all the digital domains examined. Conclusions: Frail patients are characterized by lower use of digital tools compared to robust patients, even though these patients would benefit the most from telemedicine. Digital skills were strongly influenced by frailty.

## 1. Introduction

Patients with cardiovascular (CV) conditions are typically followed up with a series of periodic examinations, but this has been severely restricted during the COVID-19 pandemic [1,2,3,4,5,6]. During the pandemic and in the immediate aftermath, healthcare providers tried to implement contact with patients through telemedicine to counterbalance the suspension of scheduled outpatient visits [7,8,9]. This included follow-up visits by phone or video calls with patients or family members to assess the clinical status and schedule a visit in case of medical need [10]. 

Among these cardiac disorders, atrial fibrillation (AF) is the most common sustained arrhythmia observed in clinical practice requiring a structured follow-up [11,12,13]. A recent survey reported a significant reduction in the activities and procedures related to arrhythmia management in Italy during the COVID-19 pandemic [1,4], highlighting the massive impact of COVID-19 on the entire organization of healthcare systems [4]. 

Telemedicine necessarily requires either the use of different digital tools (e.g., computers, webcams, and/or smartphones) and a minimum technological knowledge of the patients. These requirements may be challenging for patients with poor digital health literacy [14,15]. Digital health literacy, defined as the use of digital literacy skills to find and use health information and services, may indeed influence the use of telemedicine in most patients, particularly those with frailty [16,17]. 

The aim of our study was to explore the association between frailty, the use of digital tools, access to the Internet, and digital health literacy to determine whether it would be possible to implement telemedicine visits in patients followed in a cardiac arrhythmia outpatient clinic. 

## 2. Materials and Methods

The DIGItal health literacy after COVID-19 (DIGI-COVID) is a prospective observational single-center study conducted at the University of Modena and Reggio Emilia enrolling unselected patients referred to arrhythmia and cardiac implantable electronic device (CIED) outpatient clinics for routine follow-up. The enrollment was taken from March to September 2022. Participants were ≥18 years old and provided informed consent after detailed information was provided about the reasons for the study. The study protocol and data analysis were approved by the local ethics committee (reference number 15,758, data of approval 19/05/2021), and the research was performed in accordance with the ethical standards laid down in the 1964 Declaration of Helsinki and its later amendments. 

At enrollment, the investigators collected the following data: baseline clinical characteristics (such as age, sex, cardiovascular risk factors, and underlying heart disease), educational level (primary school, middle school, secondary school, or university), occupation (unemployed, student, employed, or retired), internet accessibility (never, at least once a month, at least once a week, more than once a week, or every day), availability of internet sources and digital tools (Wi-Fi at home, email, smartphones, tablet, and personal computers with webcam), knowledge and use of mobile apps (WhatsApp, Skype, Google Meet, Zoom, Facebook, Instagram, and Telegram), and level of confidence and propensity to use devices for AF detection (such as smartphone apps, smartwatches, or devices with a single-channel ECG). The degree of digital health literacy was assessed through the Digital Health Literacy Instrument (DHLI) scale [16]. The DHLI explores 7 digital skill categories measured by 21 self-report questions. The seven DHLI skill categories include (i) operational skills (to use the computer and Internet browser); (ii) navigation skills (to navigate and orientate on the Web); (iii) information searching (to use correct search strategies); (iv) evaluating the reliability of the information in general; (v) determining the relevance of online information; (vi) adding self-generated content to Web-based apps; and (vii) protecting and respecting privacy while using the Internet [16]. The self-reported questions require participants to rate on a 4-point scale how difficult different tasks are and how frequently they encounter certain difficulties on the Internet. The response options ranged from “never” (= 1) to “often” (= 4) or “very easy” (= 1) to “very difficult” (= 4). The total DHLI score and each skill category sub-score were calculated by summing the received scores in every single category (3 questions per each category) and then reported as mean (SD) and median (IQR). A higher score represented a lower level of digital health literacy. We also reported the number and percentages of respondents who scored 3–4 vs. 1–2 per each question. 

For the purpose of this analysis, patients were divided according to frailty status as defined by the Edmonton Frail Scale (EFS) [18]. The EFS is a valid and simple measuring instrument for identifying frailty in the clinical context [19]. The EFS is a 17-point scale that includes nine frailty domains: (i) cognition (“Clock drawing test”); (ii) general health status; (iii) functional independence; (iv) social support; (v) medication use; (vi) nutrition; (vii) mood; (viii) continence; and (ix) functional performance (“Timed Get Up and Go Test”). Consistent with previous studies, subjects with an EFS ≥ 8 were considered frail [19,20,21,22,23,24]. Specifically, we divided the population into three different categories according to the EFS score as largely applied in the literature [19,20,21,22,23,24]: (1) robust (EFS ≤ 5; (2) pre-frail (EFS 6–7), and frail patients (EFS ≥ 8). 

### Statistical Analysis

Continuous variables were expressed as median and interquartile range (IQR). Among-group comparisons were made using a non-parametric test (Kruskal–Wallis test). Categorical variables were reported as numbers and percentages. Among-groups comparisons were made using a χ2 test or Fisher’s exact test when applicable. 

A logistic regression analysis (univariate and multivariable) was performed to investigate the relationship between patients’ frailty status and non-use of the Internet (defined as never having access to the Internet). Three different multivariable logistic regression models were built: Model 1 was adjusted for age and sex, Model 2 was adjusted for comorbidities (hypertension, diabetes, previous stroke, coronary artery disease, heart failure, AF, and chronic kidney disease), and Model 3 was adjusted only for education level. The results were expressed by odds ratio (OR) and 95% confidence interval (CI). Finally, Pearson’s test was used to evaluate the correlation between EFS and DHLI. 

A two-sided *p*-value < 0.05 was considered statistically significant. All analyses were performed using SPSS statistical software (IBM Corp. Released 2019. IBM SPSS Statistics for Macintosh, Version 26.0. Armonk, NY, USA).

## 3. Results

A total of 300 patients were included in the study, of which 109 were female (36.3%). The median age of participants was 75 (66−84) years. We divided our population into three sub-groups according to frailty status evaluated using the EFS: (i) robust (EFS ≤ 5; *n* = 212, 70.7%), (ii) pre-frail (EFS 6–7; *n* = 47, 15.7%), and (iii) frail patients (EFS ≥ 8; *n* = 41, 13.7%). A detailed report of the EFS according to the three sub-groups is shown in Supplementary Table S1. Baseline characteristics stratified by frailty status are shown in Table 1 and Table 2. The degree of frailty increased with age up to 70.7% in patients over 75 years. Furthermore, 65.9% of the total females included in the study were frail (Table 1). Only a minority of patients had the highest level of education (i.e., university, *n* = 36, 12%). A downward trend from robust, pre-frail, and frail patients (14.6%, 10.6%, and 0%, respectively) was noticed. The use of digital tools and access to the Internet are shown in Table 2. The most commonly used digital tools and apps were smartphones (*n* = 223, 74.6%), emails (204/300, 68.0%), and WhatsApp (209/300, 69.7%). Pre-frail and frail patients reported a significantly lower use of emails, smartphones, PC with webcams, and WhatsApp compared to robust patients (Table 2). Interestingly, the majority of the population showed good confidence and propensity to use a device for AF detection (e.g., smartphone applications, smartwatches, and single-channel ECG) without significant differences among groups (Table 2). 

Overall, 163/300 (54.3%) patients of the total cohort had access to the Internet every day. In the univariate analysis, both pre-frail and frail patients were significantly associated with the non-use of the Internet (Table 3). After the adjustments, frailty was confirmed to be independently associated with the non-use of the Internet (Model 1 adjusted OR = 2.58, 95% CI: 1.92–5.6; Model 2 adjusted OR = 3.45, 95% CI: 1.48–8.04; and Model 3 adjusted OR = 2.54, 95% CI: 1.15–5.61) (Table 3). 

Results of the DHLI test analyzed according to frailty status are shown in Table 4. The total DHLI score was significantly higher (i.e., low digital health literacy) in pre-frail and frail patients (*p* < 0.001). Digital health literacy significantly decreased as the level of frailty increased in all the domains examined (operational skills, navigation skills, information searching, evaluating the reliability of the information, determining the relevance of online information, adding self-generated content, and protecting privacy while using the Internet) (Table 4). Pearson’s correlation showed a significant but weak positive correlation between the DHLI and EFS scores (r = 0.30, *p* < 0.001).

A detailed comparison among the study groups regarding the 21 self-reported questions is shown in Table S2. There was a progressively greater difficulty in all the digital abilities explored as the level of frailty increased. 

## 4. Discussion

In our prospective observational study, we analyzed a series of unselected patients referred to arrhythmia and CIED outpatient clinics, and we evaluated the association between the degree of frailty, the use of digital tools, and digital health literacy. 

Our findings are important for evaluating the implementation of telecardiology in routine clinical practice. Telemedicine and remote medical care started at the end of the nineteenth century, and its subsequent evolution has followed the advances in technology that occurred over the last two centuries [25,26]. The COVID-19 pandemic, due to the dramatic and profound derangement in healthcare systems, has provided a great impetus for the advancement of telemedicine, and its use has progressively increased in everyday clinical practice [27]. The benefits of telemedicine and, particularly, the potential barriers to its implementation are issues of growing concern [28].

As a reaction to the COVID-19 pandemic, the use of telemedicine in all fields of clinical activity was promoted. Implementation of telemedicine is not yet concluded since there are several problems with hospital equipment and reimbursement for the services performed [29,30]. Telemedicine can be used to make significant advancements in the management of the entire cardiovascular disease spectrum [31,32]. For example, the implementation of remote monitoring in patients with CIEDs, both for controlling the functionality of the device and for monitoring the degree of cardiac status, has been shown to reduce the use of healthcare resources without compromising patient safety [33,34,35]. In recent years, there was also a great implementation in the use of wearable devices specifically for monitoring patients with different contact methods, such as mobile apps, emails, and social media [36,37]. 

In our study, despite several challenges associated with the use of technology-related tools, patients expressed a high level of confidence and propensity to utilize these types of devices in the future, particularly those for AF detection. These data support the feasibility of performing periodic cardiological examinations in a digital way for patients with heart rhythm disturbances. Hence, the information regarding rate/rhythm control and any recurrences of AF may be controlled remotely. 

One of the main findings of our study was that frail patients were characterized by low use of digital tools and access to the Internet even though they would benefit the most from telemedicine. 

Frailty is a clinical syndrome that is frequently found in cardiology patients, characterized by reduced physiological reserve and increased vulnerability to stressors [38,39]. Several studies reported that frail patients are associated with an increased risk of adverse outcomes, thus requiring structured and holistic clinical management [38,40]. 

Frailty status together with other conditions could markedly influence the patients’ grade of digital literacy, becoming one of the most important barriers to the implementation of telemedicine [41]. Frailty indeed could negatively impact the grade of digital literacy due to the cumulative deterioration in numerous physiological systems [40,42]. Consequently, despite having the highest necessity of telemedicine, frail patients are often unable to use digital devices [43].

Our study highlighted that several digital skills, even the simplest such as using the mouse or keyboard of a computer, are markedly influenced by frailty status, supporting the need to implement digital health literacy with specific and personalized interventions in this population. We noticed indeed that performance in the DHLI decreased across all domains examined as the level of frailty increased. The domains with the worst scores were those of navigation skills, information searching, evaluating reliability, and determining the relevance of information. From this perspective, characterization of the frailty status appears to be of great clinical value in the setting of cardiology outpatients who may be potential candidates for the use of digital technologies.

In addition, frailty, despite being conceptually separate from aging, disability, and comorbidity, is indissolubly linked to these characteristics [40]. Frail patients are commonly characterized by social isolation and loneliness, and digital exclusion is still a major issue in these patients [44]. In our analysis, one-third of pre-frail and half of the frail patients answered that they never had access to the Internet, thus limiting the use of telemedicine in this population. Previous studies reported that older and frail patients who do not have access to Internet have a higher burden of comorbidities, mobility issues, and memory and attention problems [42]. 

Notably in our study, frail patients with EFS ≥ 8 were independently associated with non-use of the Internet even when the analyses were adjusted for different confounders such as sex, age, and comorbidities (Model 1 and Model 2) or only for educational level (Model 3). Other studies showed that older people with greater education levels were more likely to have basic literacy, requiring only minor assistance in using computers, resulting in high e-health literacy [45,46]. Taken together, these findings reinforce the impact of frailty on digital literacy, which is only in part influenced by age. 

The age distribution of the patients evaluated by our study is typical of a cardiological case series, with approximately half of the cases being over the age of 75, and the distribution of comorbidities is consistent with the features of patients undergoing periodic cardiological assessments [47,48]. A similar analysis was performed by our group at the same outpatient clinics two years ago during the first COVID-19 outbreak period [14]. The degree of use of digital tools was lower as compared to the present analysis [14]. We can speculate that the growing invitation to use the Internet and social media to obtain health information could explain these differences. In some cases, especially in patients with a high degree of frailty, the questionnaire has been compiled with the assistance of the caregiver explaining the difference detected over a relatively short period. In fact, the presence of social support was reported in more than 80% of the patients with frailty. Previous studies showed that older people were more likely to receive help with computer use if they were married or had social support, resulting in higher e-health literacy [45,46]. This implies that older people with social support may be able to access and benefit from telemedicine despite their frailty and lack of digital skills [42]. From this perspective, the role of the caregiver should be further implemented in this setting [49].

### Limitations of the Study

Some limitations of our study should be acknowledged. The observational nature of the study is the main limitation. However, our study has the advantage of analyzing a relatively large real-world population two years after the COVID-19 outbreak, thus providing new insights based on actual clinical practice. An inherent limitation of the study was that EFS and DHLI shared similar components, which may limit the interpretation of the results. Additionally, although the DHLI questionnaire used has several advantages such as comparability, standardization, validity, and ease of use, questionnaire-based studies have obvious inherent limitations. 

In our study, we did not differentiate the replies provided by patients and their caregivers. This could constitute a bias in the answers obtained, but it can be speculated that the degree of the frailty of the patient was reduced when a caregiver with good digital literacy was present since there was more social support. Lastly, our study is a single-center experience focused only on cardiology patients, thus limiting the generalization of the results. 

## 5. Conclusions

Our study highlighted how digital skills are strongly influenced by the frailty status of patients. Despite being the population that should benefit the most from the use of telemedicine, frail patients were characterized by a lower level of digital health literacy and use of the Internet. It is crucial to further implement effective interventions aimed at reducing this digital literacy gap. 

## Figures and Tables

**Table 1:** Baseline characteristics of the population stratified for frailty status according to the Edmonton Frail Scale.

	Robust EFS ≤ 5 (*n* = 212, 70.7%)	Pre-Frail EFS 6–7 (*n* = 47, 15.7%)	Frail EFS ≥ 8 (*n* = 41, 13.7%)	Total *n* = 300	*p*
**Age**, median (IQR)	73 (63–82)	82 (72–86)	84 (74–88)	75 (66–84)	<0.001
**Age classes**, *n* (%)					<0.001
*<65*	58/212 (27.4)	8/47 (17.0)	1/41 (2.4)	67/300 (22.3)	
*65–75*	69/212 (32.5)	6/47 (12.8)	11/41 (26.8)	86/300 (28.7)	
*>75*	85/212 (40.1)	33/47 (70.2)	29/41 (70.7)	147/300 (49.0)	
**Female**, *n* (%)	66/212 (31.1)	16/47 (34.0)	27/41 (65.9)	109/300 (36.3)	<0.001
**Site of enrollment**, *n* (%)					0.69
*Arrhythmological clinic*	106/212 (50.0)	26/47 (55.3)	19/41 (46.3)	151/300 (50.3)	
*CIED clinic*	106/212 (50.0)	21/47 (44.7)	22/41 (53.7)	149/300 (49.7)	
**Obesity**, *n* (%)	42/212 (19.8)	8/47 (17.0)	7/41 (17.1)	57/300 (19.0)	0.750
**Hypertension**, *n* (%)	157/212 (74.1)	41/47 (87.2)	39/41 (95.1)	237/300 (79.0)	0.003
**Diabetes**, *n* (%)	38/212 (17.9)	12/47 (25.5)	12/41 (29.3)	62/300 (20.7)	0.17
**Smoking** (current), *n* (%)	22/212 (10.4)	2/47 (4.3)	1/41 (2.4)	25/300 (8.3)	0.17
**Dyslipidaemia**, *n* (%)	127/212 (59.9)	28/47 (59.6)	20/41 (48.8)	175/300 (58.3)	0.41
**Previous stroke/TIA**, *n* (%)	14/212 (6.6)	5/47 (10.6)	7/41 (17.1)	26/300 (8.7)	0.08
**Coronary artery disease**, *n* (%)	51/211 (24.2)	16/47 (34.0)	15/41 (36.6)	82/299 (27.4)	0.14
**Heart failure**, *n* (%)	55/212 (25.9)	19/47 (40.4)	6/40 (15.0)	80/299 (26.8)	0.02
**Atrial fibrillation**, *n* (%)	138/211 (65.4)	37/47 (78.7)	31/41 (75.6)	206/299 (68.9)	0.12
**Valvular heart disease**, *n* (%)	27/212 (12.7)	10/47 (21.3)	11/41 (26.8)	48/300 (16.0)	0.04
**Dilated cardiomyopathy**, *n* (%)	38/212 (17.9)	12/47 (25.5)	2/40 (5.0)	52/299 (17.4)	0.03
**COPD**, *n* (%)	16/212 (7.5)	6/47 (12.8)	4/41 (9.8)	26/300 (8.7)	0.49
**Chronic kidney disease**, *n* (%)	19/212 (9.0)	14/47 (30.4)	12/41 (29.3)	45/300 (15.0)	<0.001
**Thyroid dysfunction**, *n* (%)	24/212 (11.3)	10/47 (21.3)	11/41 (26.8)	45/300 (15.0)	0.017
**Active cancer**, *n* (%)	15/212 (7.1)	5/47 (10.6)	9/41 (22)	29/300 (9.7)	0.045
**Liver disease**, *n* (%)	6/212 (2.8)	2/47 (4.3)	4/41 (9.8)	12/300 (4.0)	0.116
**Educational level**, *n* (%)					0.002
*Primary*	64/212 (30.2)	21/47 (44.7)	25/41 (61.0)	110/300 (36.6)	
*Middle*	55/212 (25.9)	14/47 (29.8)	8/41 (19.5)	77/300 (25.7)	
*Secondary*	62/212 (29.2)	7/47 (14.9)	8/41 (19.5)	77/300 (25.7)	
*University*	31/212 (14.6)	5/47 (10.6)	0/41 (0)	36/300 (12.0)	
**Occupational Level**, *n* (%)					<0.001
*Unemployed*	4/212 (1.9)	3/47 (6.4)	0/41 (0)	7/300 (2.3)	
*Employed*	50/212 (23.6)	4/47 (8.5)	0/41 (0)	54/300 (18.0)	
*Retired*	158/212 (74.5)	40/47 (85.1)	41/41 (100)	239/300 (79.7)	

CIED, cardiac implantable electronic device; COPD, chronic obstructive pulmonary disease; EFS, Edmonton Frail Scale; IQR, interquartile range; TIA, transient ischemic attack. Valvular heart disease was defined as a history of at least moderate regurgitation or stenosis or prior valve surgery.

**Table 2:** Characteristics of Internet access and use of digital tools stratified for frailty status according to the Edmonton Frail Scale.

	Robust EFS ≤ 5 (*n* = 212, 70.7%)	Pre-Frail EFS 6–7 (*n* = 47, 15.7%)	Frail EFS ≥ 8 (*n* = 41, 13.7%)	Total *n* = 300	*p*
**Internet Access**, *n* (%)					0.002
*Never*	43/212 (20.3)	17/47 (36.2)	21/41 (51.2)	81/300 (27.0)	
*At least 1/month*	2/212 (0.9)	1/47 (2.1)	0/41 (0)	3/300 (1.0)	
*At least 1/week*	11/212 (5.2)	4/47 (8.5)	0/41 (0)	15/300 (5.0)	
*More than 1/week*	32/212 (15.1)	3/47 (6.4)	3/41 (7.3)	38/300 (12.7)	
*Everyday*	124/212 (58.5)	22/47 (46.8)	17/41 (41.5)	163/300 (54.3)	
**Wi-Fi at home**, *n* (%)	143/212 (67.5)	23/47 (48.9)	23/40 (57.5)	189/299 (63.2)	0.04
**Email**, *n* (%)	154/212 (72.6)	29/47 (61.7)	21/41 (51.2)	204/300 (68.0)	0.01
**Device use**, *n* (%)					
*Smartphone*	171/212 (80.7)	28/47 (59.6)	24/40 (60.0)	223/299 (74.6)	0.001
*Tablet*	68/212 (32.1)	18/47 (38.3)	10/41 (24.4)	96/300 (32.0)	0.37
*PC with webcam*	118/212 (55.7)	17/47 (36.2)	12/41 (29.3)	147/300 (49.0)	0.001
**Application use**, *n* (%)					
*WhatsApp*	162/212 (76.4)	26/47 (55.3)	21/41 (51.2)	209/300 (69.7)	<0.001
*Skype*	32/212 (15.1)	8/47 (17.0)	5/41 (12.2)	45/300 (15.0)	0.81
*Google Meet*	47/212 (22.2)	7/47 (14.9)	9/41 (22.0)	63/300 (21.0)	0.53
*Zoom*	45/212 (21.2)	7/47 (14.9)	10/41 (24.4)	62/300 (20.7)	0.51
*Facebook*	99/212 (46.7)	19/47 (40.4)	13/41 (31.7)	131/300 (43.7)	0.18
*Instagram*	63/212 (29.7)	12/47 (25.5)	10/41 (24.4)	85/300 (28.3)	0.70
*Telegram*	22/212 (10.4)	1/47 (2.1)	3/41 (7.3)	26/300 (8.7)	0.18
**Device for AF detection use**, *n* (%)					
*Confidence*	173/212 (81.6)	37/47 (78.7)	29/41 (70.7)	239/300 (79.7)	0.28
*Propensity*	169/212 (79.7)	35/47 (74.5)	29/41 (70.7)	233/300 (77.7)	0.38

AF, atrial fibrillation; EFS, Edmonton Frail Scale.

**Table 3:** Association between frailty and non-use of the Internet.

	Unadjusted			Model 1			Model 2			Model 3		
	OR	95% CI	*p*	aOR	95% CI	*p*	aOR	95% CI	*p*	aOR	95% CI	*p*
**Robust** *(ref)*	**-**	**-**	**-**	**-**	**-**	**-**	**-**	**-**	**-**	**-**	**-**	**-**
**Pre-frail**	**2.22**	**1.12–4.41**	**0.02**	1.44	0.68–3.03	0.33	1.37	0.62–3.02	0.42	1.78	0.81–3.89	0.14
**Frail**	**4.12**	**2.05–8.29**	**<0.001**	**2.58**	**1.92–5.61**	**0.01**	**3.45**	**1.48–8.04**	**0.004**	**2.54**	**1.15–5.61**	**0.02**

Model 1 was adjusted for age and sex; Model 2 was adjusted for age, sex, and comorbidities (hypertension, diabetes, previous stroke, coronary artery disease, heart failure, atrial fibrillation, and chronic kidney disease). Model 3 was adjusted only for educational level. aOR, adjusted odds ratio; CI, confidence interval; OR, odds ratio.

**Table 4:** Digital Health Literacy Instrument according to frailty status.

	Robust EFS ≤ 5 (*n* = 212, 70.7%)	Pre-Frail EFS 6–7 (*n* = 47, 15.7%)	Frail EFS ≥ 8 (*n* = 41, 13.7%)	Total *n* = 300	*p*
**Total digital health literacy**					<0.001
Median (IQR)	36 (27–58)	45 (32–84)	84 (38–84)	38 (29–84)	
Mean (SD)	44.86 (22.76)	53.94 (25.53)	61.66 (24.51)	48.58 (24.16)	
**Operational skills**					<0.001
Median (IQR)	3 (3–9)	6 (3–12)	12 (3–12)	3 (3–12)	
Mean (SD)	5.81 (3.71)	7.53 (4.24)	8.32 (4.29)	6.42 (3.99)	
**Navigation skills**					<0.001
Median (IQR)	6 (4–10)	7 (5–12)	12 (7–12)	6 (4–12)	
Mean (SD)	6.73 (3.36)	8.02 (3.40)	9.46 (2.99)	7.30 (3.45)	
**Information searching**					0.002
Median (IQR)	6 (3–9)	6 (3–12)	12 (6–12)	6 (3–12)	
Mean (SD)	6.46 (3.42)	7.49 (3.85)	8.73 (3.71)	6.93 (3.61)	
**Evaluating reliability**					<0.001
Median (IQR)	6 (4–9)	6 (5–12)	12 (6–12)	6 (4–12)	
Mean (SD)	6.75 (3.33)	7.77 (3.59)	9.05 (3.34)	7.22 (3.45)	
**Determining relevance**					0.005
Median (IQR)	6 (3–12)	6 (3–12)	12 (6–12)	6 (3–12)	
Mean (SD)	6.80 (3.46)	7.55 (3.90)	8.90 (3.60)	7.21 (3.61)	
**Adding self-generated content**					<0.001
Median (IQR)	5 (3–9)	6 (3–12)	12 (5–12)	6 (3–12)	
Mean (SD)	6.21 (3.67)	7.64 (3.91)	8.63 (3.78)	6.76 (3.81)	
**Protecting privacy**					<0.001
Median (IQR)	4 (3–12)	9 (3–12)	12 (5–12)	5 (3–12)	
Mean (SD)	6.10 (3.71)	7.94 (4.07)	8.56 (3.87)	6.73 (3.90)	

EFS, Edmonton Frail Score; IQR, interquartile range; SD, standard deviation.

## Data Availability

The data present in this study are not publicly available but are available on reasonable request to the corresponding author.

## Supplementary Materials

The following supporting information can be downloaded at: https://www.mdpi.com/article/10.3390/jpm13010099/s1, Table S1: Edmonton Frail Scale; Table S2: Difficulty in using the internet calculated according to the Digital Heath Literacy Instrument stratified by frailty status.
