# Supplementary material for: DIGItal Health Literacy after COVID-19 Outbreak among Frail and Non-Frail Cardiology Patients: The DIGI-COVID Study
Source: J Pers Med. 2022 Dec 31;13(1):99. doi: 10.3390/jpm13010099 (PMC9863916; doi:10.3390/jpm13010099)
Supplement: Supplementary file 1 [file jpm-13-00099-s001.zip › jpm-2108142-supplementary.pdf]

SUPPLEMENTARY MATERIALS

DIGItal health literacy after COVID-19 outbreak among frail and non-frail cardiology patients: the DIGI-COVID study

Marco Vitolo [1,2], Valentina Ziveri [1], Giacomo Gozzi [1], Chiara Busi [1], Jacopo Francesco Imberti [1,2], NiccolòBonini [1,2], Federico Muto [1], Davide Antonio Mei [1], Matteo Menozzi [1], Marta Mantovani [1], Benedetta Cherubini [1], Vincenzo Livio Malavasi [1] and Giuseppe Boriani [1,*]

Table S1. Edmonton Frail Scale

Table S2. Difficulty in using the internet calculated according to the Digital Heath Literacy Instrument stratified by frailty status.

Table S1. Edmonton Frail Scale

FRAILTY DOMAIN	ITEM	**Robust** EFS ≤ 5 (n=212, 70.7%)	**Pre-frail** EFS 6-7 (n=47, 15.7%)	**Frail** EFS ≥8 (n=41, 13.7%)	**Total** (N=300)
**Cognition**					
	No errors	153/212 (72.2)	17/47 (36.2)	4/41 (9.8)	174/300 (58.0)
	Minor errors	55/212 (25.9)	22/47 (46.8)	26/41 (63.4)	103/300 (34.3)
	Other errors	4/212 (1.9)	8/47 (17)	11/41 (26.8)	23/300 (7.7)
**General health status**					
Hospital admission					
	0	88/212 (41.5)	7/47 (14.9)	10/41 (24.4)	105/300 (35.0)
	1-2	103/212 (48.6)	27/47 (57.4)	9/41 (22)	139/300 (46.3)
	>2	21/212 (9.9)	13/47 (27.7)	22/41 (53.7)	56/300 (18.7)
Self-perceived health status					
	Excellent/good	111/212 (52.4)	7/47 (14.9)	0/41 (0)	118/300 (39.3)
	Fair	89/212 (42)	27/47 (57.4)	23/41 (56.1)	139/300 (46.3)
	Poor	12/212 (5.7)	13/47 (27.7)	18/41 (43.9)	43/300 (14.3)
**Functional indipendence**					
(n activities non-self-efficient)	0-1	202/212 (95.3)	34/47 (72.3)	13/41 (31.7)	249/300 (83.0)
	2-4	10/212 (4.7)	12/47 (25.5)	16/41 (39)	38/300 (12.7)
	5-8	0/212 (0)	1/47 (2.1)	12/41 (29.3)	13/300 (4.3)
**Social support**					
	Always	198/212 (93.4)	41/47 (87.2)	34/41 (82.9)	273/300 (91.0)
	Sometimes	13/212 (6.1)	4/47 (8.5)	6/41 (14.6)	23/300 (7.7)
	Never	1/212 (0.5)	2/47 (4.3)	1/41 (2.4)	4/300 (1.3)
**Medication use**					
Five ore more medications	Yes	127/212 (59.9)	42/47 (89.4)	39/41 (95.1)	208/300 (69.3)
Forget to take medications	Yes	26/212 (12.3)	12/47 (25.5)	13/41 (31.7)	51/300 (17.0)
**Nutrition** *(weight loss)*	Yes	15/212 (7.1)	10/47 (21.3)	12/41 (29.3)	37/300 (12.3)
**Mood depressed**	Yes	24/212 (11.3)	20/47 (42.6)	29/41 (70.7)	73/300 (24.3)
**Incontinence**	Yes	11/212 (5.2)	5/47 (10.6)	16/41 (39)	32/300 (10.7)
**Functional performance** *(Timed Up and Go)*					
	0-10 sec	161/212 (75.9)	14/47 (29.8)	5/41 (12.2)	180/300 (60.0)
	11-20 sec	46/212 (21.7)	27/47 (57.4)	11/41 (26.8)	84/300 (28.0)
	>20 sec	5/212 (2.4)	6/47 (12.8)	25/41 (61)	36/300 (12.0)

Legend: EFS, Edmonton Frail Scale.

Table S2. Difficulty in using the internet calculated according to the Digital Heath Literacy Instrument stratified by frailty status.

		**Robust** EFS ≤ 5 (n=212, 70.7%)	**Pre-Frail** EFS 6-7 (n=47, 15.7%)	**Frail** EFS ≥8 (n=41, 13.7%)	**Total** (N=300)	*p*
**Operational skills**	**How difficult/very difficult is it for you to…**					
	Use the keyboard of a computer (eg, to type words)?	57/212 (26.9)	22/47 (46.8)	23/41 (56.1)	102/300 (34.0)	<0.001
	Use the mouse (eg, to use the cursor or to click)?	56/212 (26.4)	22/47 (46.8)	23/41 (56.1)	101/300 (33.7)	<0.001
	Use the buttons or links on websites?	58/212 (27.4)	22/47 (46.8)	23/41 (56.1)	103/300 (34.3)	<0.001
**Information searching**	**When you search the Internet for information on health, how difficult/very difficult is it for you to…**					
	Make a choice from all the information you find?	70/212 (33.0)	20/47 (42.6)	26/41 (63.4)	116/300 (38.7)	0.001
	Use the proper words or search query to find the information you are looking for?	63/212 (29.7)	20/47 (42.6)	24/41 (58.5)	107/300 (35.7)	0.001
	Find the exact information you are looking for?	63/212 (29.7)	20/47 (42.6)	26/41 (63.4)	109/300 (36.3)	<0.001
**Evaluating reliability**	Decide whether the information is reliable or not?	122/212 (57.5)	28/47 (59.6)	34/41 (82.9)	184/300 (61.3)	0.009
	Decide whether the information is written with commercial interests?	64/212 (30.2)	20/47 (42.6)	23/41 (56.1)	107/300 (35.7)	0.004
	Check different websites to see whether they provide the same information?	82/211 (38.9)	22/47 (46.8)	28/41 (68.3)	132/299 (44.1)	0.002
**Determining relevance**	Decide if the information you found is applicable to you?	72/212 (34.0)	21/47 (44.7)	26/41 (63.4)	119/300 (39.7)	0.001
	Apply the information you found in your daily life?	75/212 (35.4)	22/47 (46.8)	26/41 (63.4)	123/300 (41.0)	0.003
	Use the information you found to make decisions about your health?	76/212 (35.8)	21/47 (44.7)	27/41 (65.9)	124/300 (41.3)	0.001
**Navigation skills**	**When you search the Internet for health information, how often/very often does it happen that…**					
	You lose track of where you are on a website or the Internet?	58/212 (27.4)	18/47 (38.3)	25/41 (61.0)	101/300 (33.7)	<0.001
	You do not know how to return to a previous page?	79/212 (37.3)	23/47 (48.9)	32/41 (78.0)	134/300 (44.7)	<0.001
	You click on something and get to see something different than you expected?	114/212 (53.8)	31/47 (66.0)	35/41 (85.4)	180/300 (60.0)	0.001
**Adding self-generated content**	**When typing a message (to your doctor, on a forum, on social media) how difficult/very difficult is it for you to…**					
	Clearly formulate your question or health-related worry?	64/212 (30.2)	21/47 (44.7)	24/41 (58.5)	109/300 (36.3)	0.001
	Express your opinion, thoughts, or feelings in writing?	65/212 (30.7)	21/47 (44.7)	24/41 (58.5)	110/300 (36.7)	0.001
	Write your message as such, for people to understand exactly what you mean?	63/212 (29.7)	21/47 (44.7)	24/41 (58.5)	108/300 (36.0)	0.001
**Protecting privacy**	**When you post a message on a public forum or social media, how often/very often…**					
	Do you find it difficult to judge who can read along?	65/212 (30.7)	24/47 (51.1)	23/41 (56.1)	112/300 (37.3)	0.001
	Do you (intentionally or unintentionally) share your own private information?	61/212 (28.8)	24/47 (51.1)	22/41 (53.7)	107/300 (35.7)	0.001
	Do you (intentionally or unintentionally) share some else's private information?	62/212 (29.2)	24/47 (51.1)	22/41 (53.7)	108/300 (36.0)	0.001

Legend: EFS, Edmonton Frail Score. The table reports the numbers and percentages of respondents who answered 3 or 4 per each question.
